# The effects of cognitive behavioural therapy on depression and quality of life in patients with maintenance haemodialysis: a systematic review

**DOI:** 10.1186/s12888-020-02754-2

**Published:** 2020-07-14

**Authors:** Chen Ling, Debra Evans, Yunfang Zhang, Jianying Luo, Yanping Hu, Yuxia Ouyang, Jiamin Tang, Ziqiao Kuang

**Affiliations:** 1 Department of Nephrology, Huadu Hospital, Southern Medical University (People’s Hospital of Huadu District), 22 Baohua Road, Huadu District, Guangzhou, 510800 People’s Republic of China; 2 The Third School of Clinical Medicine, Southern Medical University, Guangzhou, People’s Republic of China; 3 Birmingham City University, City South Campus, Faculty of Health, Education and Life Sciences, 15 Bartholomew Row, Birmingham, B5 5JU UK; 4 Nursing Department, Huadu Hospital, Southern Medical University (People’s Hospital of Huadu District), 48 Xinhua Road, Huadu District, Guangzhou, 510800 People’s Republic of China; 5 Department of Breast Surgery, Huadu Hospital, Southern Medical University (People’s Hospital of Huadu District), 48 Xinhua Road, Huadu District, Guangzhou, 510800 People’s Republic of China

**Keywords:** Haemodialysis, Cognitive behavioural therapy, Depression, Quality of life

## Abstract

**Background:**

Depression is highly prevalent among Haemodialysis (HD) patients and is known to results in a series of adverse outcomes and poor quality of life (QoL). Although cognitive behavioural therapy (CBT) has been shown to improve depressive symptoms and QoL in other chronic illness, there is uncertainty in terms of the effectiveness of CBT in HD patients with depression or depressive symptoms.

**Methods:**

All randomised controlled trials relevant to the topic were retrieved from the following databases: CINHAL, MEDLINE, PubMed, PsycINFO and CENTRAL. The grey literature, specific journals, reference lists of included studies and trials registers website were also searched. Data was extracted or calculated from included studies that had measured depression and quality of life using valid and reliable tools –this included mean differences or standardised mean differences and 95% confidence intervals. The Cochrane risk of bias tool was used to identify the methodological quality of the included studies.

**Results:**

Six RCTs were included with varying methodological quality. Meta-analysis was undertaken for 3 studies that employed the CBT versus usual care. All studies showed that the depressive symptoms significantly improved after the CBT. Furthermore, CBT was more effective than usual care (MD = − 5.28, 95%CI − 7.9 to − 2.65, *P* = 0.37) and counselling (MD = − 2.39, 95%CI − 3.49 to − 1.29), while less effective than sertraline (MD = 2.2, 95%CI 0.43 to 3.97) in alleviating depressive symptoms. Additionally, the CBT seems to have a beneficial effect in improving QoL when compared with usual care, while no significant difference was found in QoL score when compared CBT with sertraline.

**Conclusions:**

CBT may improve depressive symptoms and QoL in HD patients with comorbid depressive symptoms. However, more rigorous studies are needed in this field due to the small quantity and varied methodological quality in the identified studies.

## Background

End stage renal disease (ESRD) is a leading cause of morbidity and mortality worldwide, and it has a sharply increasing incidence and prevalence. Globally, the number of ESRD patients was 2.62 million in 2010 [1] and it is predicted to increase to more than double by 2030 to 5.4 million [2]. The increased ESRD prevalence is predominantly due to the incidence of diabetes and hypertension stay high and show an increasing trend [3]. Currently, HD is the mainstream treatment for ESRD patients, and 90% of them are receiving this therapy worldwide [4].

Depression is a prominent psychological problem in HD patients. It is estimated that HD patients have an approximately four-fold incidence of depression compared to the general population [5]. A multinational cross-sectional study found that the prevalence of depression was up to 46% from 2278 HD participants [6]. The depression symptoms of HD patients are associated with a series of adverse outcomes, for instance, lower treatment compliance [7, 8], malnutrition, increased morbidity [9], decreased quality of life, higher rates of hospitalisation and mortality among HD patients [10–12]. However, depression issues are often under recognized and untreated [13]. Therefore, these severe outcomes indicated the importance of monitoring the mental state of the patients as well as the necessity of providing effective treatments for patients with HD.

CBT is one of the most widely practised therapeutic approaches in psychology. CBT reduces depressive symptoms by identifying inaccurate and maladaptive cognitions, testing the cognitions against reality, and modifying the dysfunctional thoughts, emotions and behaviours through different strategies accordingly [14]. The standard techniques of CBT which are utilised in treating depression are divided into two parts. The cognitive techniques include cognition identification, thought recording, cognition restructuring, thought testing and distraction strategy training [15, 16]. The behavioural techniques consist of goal setting, activity scheduling, relaxation training and relapse prevention [17].

NICE clinical guideline [18] recommended CBT as a therapy for depression in people with chronic diseases. Subsequently, growing evidence has been shown that CBT is a well-established intervention in depression in different chronic diseases, such as diabetes, hypertension, heart failure co-morbid depression patients [19–21]. It also has a promising effect on some patients’ QoL. However, the effects of CBT on HD patients with depression remains unclear because there is no systematic review that specifically targets this issue.

Previously, there were three systematic reviews [22–24] that investigated the effects of psychological therapies on depression in HD and Chronic kidney disease patients. While these reviews included CBT studies, due to small quantity of the included articles of CBT and the included patients were not required to be assessed by the validated depression scales, there is a lack of conclusion which specifically emphasises the effect of CBT. The authors of the systematic reviews also recommended that certain types of psychological interventions could be investigated to reach more reliable conclusions [22]. Given that new RCTs have emerged after these three systematic reviews, there is a need to upgrade the evidence to assess the impact of CBT on patients’ reported measures of depression and QoL in individuals with HD.

In the present systematic review, randomised controlled trials (RCTs) were included exclusively. A randomised controlled trial is a type of scientific experiment that randomly allocating subjects to two or more groups, treating them differently, and then comparing them with respect to a measured response. Due to the randomised allocating process, this type of trial can reduce certain sources of bias, such as selection bias, when testing the effectiveness of treatments.

## Methods

This article adherences to the PRISMA guidelines [25] for systematic review. The PRISMA checklist for this systematic review is presented in Additional file 1 (supplementary material).

### Criteria for considering studies for this systematic review

#### The type of studies conducted

Randomised controlled trials.

#### The type of participants involved

Participants were limited to adult patients (aged 18 years and over) with HD treatment (more than 3 months) and depression or depressive symptoms. Studies were included if participants who had depression or depressive symptoms were assessed by investigators using structured clinic interview (DSM) or validated depression scales. Studies whose patients had cognitive dysfunction were excluded because they could not understand and follow the procedures of CBT.

#### The type of interventions and comparison intervention used

The intervention of interest in this systematic review was CBT or CBT-based intervention. The included studies had to entail both cognitive and behavioural components, such as cognitive restructuring, behavioural activation, muscle relaxation and deep breathing. Studies which solely comprise cognitive therapy or behavioural therapy were excluded because they did not belong to the definition of CBT.

The intervention in included articles was CBT conducted by therapists or professional nurse or in a computerised CBT. The formats of CBT could be delivered individually (by telephone or face-to-face) or in groups. The comparison interventions could include no treatment, usual care, waiting lists and any other therapies.

#### The type of outcome measured

The outcomes of interest in this systematic review were depression and QoL among HD patients. There was no limitation on the types of validated scales relevant to depression and QoL.

#### Language, full-text availability and the timeline of the studies

Studies included in this review were required to be the English language and full-text articles. Only studies undertaken from January 1976 were included in this systematic review. According to Silverstein [26], thrice-weekly HD treatment has over four decades of routine access and clinical experience for adult HD patients. This means that the regular maintenance HD was started in 1976. The history of CBT can be traced back to the 1960s [27], which was longer than the maintenance HD treatment. Therefore, the present author identified the search dates range from January 1976 to July 2019.

### Search strategy

#### Electronic database search

Index term, such as Medical Subject Headings (MeSH) and free texts were used to ensure a comprehensive and specific search. The identified key search terms were “haemodialysis”, “cognitive behaviour therapy”, “cognitive therapy”, “behavioural therapy” and “depression”. The corresponding synonyms, abbreviations and truncations were utilised to expand the search range also. The full electronic search strategy is presented in Additional file 2 (supplementary material).

The following electronic databases were visited to identify the relevant RCTs: CINHAL, MEDLINE, PsycINFO, PubMed, CENTRAL (from 1st April 2019 up to 4th July 2019). The search record of CINHAL is attached in Additional file 3 (supplementary material)

#### Complementary search

The present author searched some specialist journals, such as *Journal of Renal Care*; *BMC Nephrology; International Urology and Nephrology; American Journal of Kidney Diseases; Hemodialysis International.* Also, the present author browsed the reference lists of relevant systematic reviews and all included studies to identify additional articles that might have been missed from an electronic search.

##### Grey literature

To find as much evidence as possible, http://ethos.bl.uk/, www.opengrey.eu/ and https://scholar.google.com/ were searched to identify relevant dissertations, conference abstracts or other research papers.

To ascertain the conclusions of the systematic review were as up to date as possible, the present author searched the trials registers website, such as www.ClinicalTrials.gov.

### Study selection procedures

There were two stages of selection work. The first stage was reviewing the title and abstract. Initially, all the search results from different databases were downloaded into Endnote Version 9.0 software. Duplicate literature records were removed by the software. Then, all the titles and abstracts of the imported literature were scanned by the present author. The standard of the reviewing was based on the population, intervention, comparative intervention, outcome and type of study. Articles that were not relevant to the topic of the systematic review were excluded. For those articles that met the inclusion criteria, or they did not provide enough information in the abstract, the full-text articles were required. If the full text of research could not be obtained after contacting the article author, applying for the inter-library loans service, or using any other methods, the articles were excluded. Those obtained full-text articles were brought into the next stage of selection.

The second stage was reviewing the full-text paper. The standard of the reviewing was based on the inclusion criteria and exclusion criteria. For the studies which could not be determined by the author, they were discussed with the second author to achieve a consensus result. The selection of articles was followed with the PRISMA flowcharts and presented with a diagram.

### Quality assessment

The Cochrane risk of bias tool was used to assess the potential bias in the studies included in the present systematic review. Included studies were assessed via six domains, including selection bias, performance bias, detection bias, attrition bias, reporting bias and other bias. The results of the assessment were expressed as low bias risk, high bias risk and unclear bias risk. RevMan 5.3 software was used to present the results of the quality assessment more visually.

### Data extraction

A pre-designed data extraction form was employed to collect relevant and necessary information of included studies. The data to be extracted include details of study information (authors, published year country and publication), methods (aims of the study, study design, setting), participants (sample size and allocation, drop out, mean age, gender, inclusion criteria, and exclusion criteria), interventions (including descriptions of the implementation process of CBT and counter-intervention, frequency and length of intervention, length of follow-up, amount of contact, adverse effects and deliverers), outcomes (primary and secondary outcomes specified and collected), results (the depression and QoL scores at baseline, post-intervention and follow-up), conclusions and the results of the assessment of the risk of biases.

### Data synthesis

In this systematic review, the included comparison interventions were usual care, no intervention and any other therapies. Due to the diversity of interventions included, narrative synthesis combined with meta-analyses may be used in the present review. To measure the clinical effectiveness of the intervention, mean differences (MD) and the corresponding 95% confidence intervals (CI) were calculated. To assess the heterogeneity among studies, chi-square test and I^2^ were utilised. If the tested heterogeneity is not significant (*P* ≥ 0.1, I^2^ ≤ 50), the fixed-effect model can be used. If the tested heterogeneity is distinct (*P*<0.1, I^2^>50), the random effect model can be used in meta-analysis [28]. The amount of heterogeneity was evaluated visually by conducting a forest plot [29].

## Results

### Results of the search strategy

**Fig. 1:**
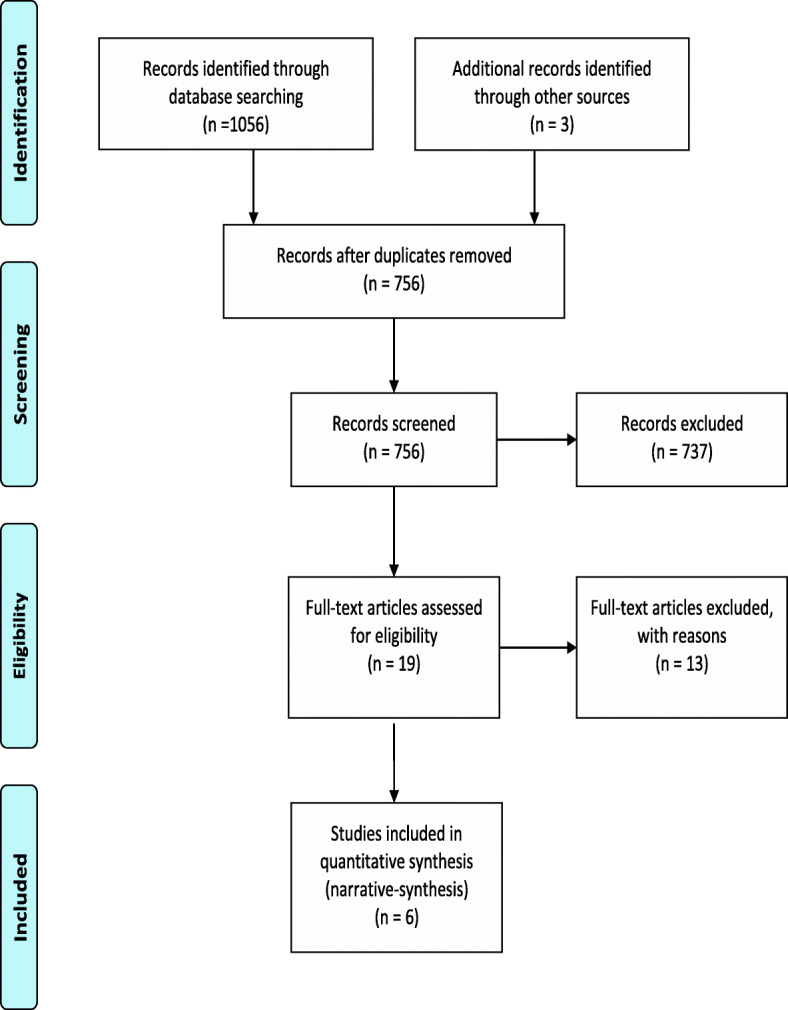
PRISMA Flowchart for search result. Detailed legend: The initial search of electronic databases yielded a total of 1056 records, and 3 records were identified through other resources. After the removal of duplicate studies and careful appraisal of titles, abstracts and full-text, 6 articles were included in the present systematic review

The initial search of electronic databases yielded a total of 1056 records, and 3 records were identified through other resources. After the removal of duplicate studies and careful appraisal of titles, abstracts and full-text, 6 articles were included in the present systematic review. The process of literature retrieval is summarised in Fig. 1 below. The characteristics of excluded studies are summarised in Additional file 4 (supplementary material).

### Characteristics of included studies

A total of six RCTs and 479 participants were included in the current review (248 in CBT groups, 231 in control groups). The studies all published between 2009 and 2019. The sample sizes ranging from 49 to 116 patients per study. In this population, 51.6% of the participants were males whilst 48.4% of them were females. Studies specifically recruited adult patients over 18 years old, and the mean age of this population ranged from 41.7 to 54.0.

All studies included HD participants with depressive symptoms, while with different criteria. The inclusion criteria, characteristics of the population and baseline are summarised in Table 1 below. According to the scoring instructions of different depression scales and the baseline depression score, the included participants were assessed as mild to moderate depression before the treatment in Lerma et al.’s study [30]; moderate depression in four studies [31–34]; and moderate to severe depression in Al saraireh et al.’s study [35]. The depressive symptoms in above studies were measured by the Beck Depression Inventory (BDI), Hamilton Depression Rating Scale (HDRS), Mini International Neuropsychiatric Interview (MINI), Hospital Anxiety and Depression Scale (HADS) and Quick Inventory of Depressive Symptoms-Clinician-rated (QIDS-C).

**Table 1:** Characteristics of study design, inclusion criteria, population and baseline

Study ID	Study design	Inclusion criteria	Sample size (I/C), male %	Mean age	Dropouts	Baseline depression score Mean (SD)	
Duarte (2009) [31]	RCT	age:18-80 HD>3 months Mini International Neuropsychiatric Interview≥5	85 (41/44) Male: 35 (38.9%)	I: (52.4±15.9), C: (54.0±12.7)	5 I:5	BDI I: 24.2 (9.7) C: 27.3 (10.7)	
Cukor (2014) [32]	RCT	age>18 HD>6 months depression scale BDI-II >10	59 (33/26) Male: 16 (27%)	Not reported	6 Not reported the detailed dropout rates in each group	BDI-II I: 24.7 (9.8) C: 21.9 (8.9)	HAM-D I: 15.7 (6.8) C: 12.9 (5.3)
Lerma (2017) [30]	RCT	age>18 HD>6 months BDI: mild or moderate scores	49 (31/18) Male: 23 (47%)	I: **(**41.8±14.7) C:(41.7±15.1)	11 I:7 C:4	BDI I: 13.6 (7.6) C: 15.8 (10.0)	
Valsara (2016) [33]	RCT	age:20-65 HD>1 year HADS score>7	67 (33/34) Male: 47 (70.2%)	66.67% in 43 to 65 years of age,	13	HADS I: 11.85 (2.15) C: 11.21 (2.53)	
Mehrotra (2019) [34]	RCT	age≥21 HD ≥3 months BDI-II score≥15	114 (56/58) Male: 68 (57%)	I: (50±13), C:(53±12)	6 I:45 C:2	QIDS-C I: 12.2 (5.1) C: 10.9 (4.9)	
Al saraireh (2018) [35]	RCT	HD>1 year Hamilton depression rating scale	105 (51/54) Male: 52 (50%)	I: (53.4±8.0) C:(52±10.7)	25 I: 11 C:14	HAM-D I: 19.5 (5.4) C: 19.6 (5.4)	

*I* intervention group, *C* comparison

#### Details of study interventions and comparisons

All the intervention groups included both the cognitive and behaviour elements. Moreover, all of the studies used a face-to-face method to conduct CBT. However, these CBT were varied in format, delivery and duration. In four studies, the CBT interventions were conducted by individual format [32–35]. The remaining two studies evaluated group CBT programmes, each group consisting of 3–6 patients [30, 31]. Overall, the duration of CBT varied from 5 weeks to 12 weeks, and the study follow-up period ranged from 1 month to 6 months after the post-treatment. Each weekly session lasted 1 h to 2 h. The interventions were delivered by psychologists, therapists without description, or nurses who had CBT expertise.

In the comparison groups, three studies compared CBT against usual care (also sometimes described in trials as treatment as usual or waiting list) [30–32]. The remaining three studies compared CBT with active comparisons groups comprising counselling [33], psychoeducation [35] and antidepressants [34]. Table 2 provides the detailed characteristics of the included studies below.

**Table 2:** Characteristics of the included studies

Study ID	Intervention group	Comparison group	Outcome	Measures	Follow up
Duarte (2009) [31]	Group CBT:12 weekly sessions (4 participants per group) 1 hour each session (1) self-monitoring of mood status (2) cognitive restructuring (3) pleasant activities (4) social abilities (5) relaxation exercises with positive imagination Delivered by a licenced psychologist	Usual care	depression QoL	BDI MINI KDQOL-SF	6 months after treatment
Cukor (2014) [32]	Individual chairside CBT:12 weekly sessions 1 hour each session (1) assessment (2) psychoeducation of depression and medical illness (3) behavioural activation, (4) cognitive intervention Delivered by a doctoral-level psychologist	Usual care (waiting list)	depression QoL	BDI-II HAM-D KDQOL-SF	3 months after treatment
Lerma (2017) [30]	Group CBT: 5 weekly sessions (3-6 participants per group) 2 hours each session (1) Behavioural activation (2) Deep breathing and muscle relaxation (3) Cognitive restructuring Delivered by: Therapist	Usual care (waiting list)	depression QoL	BDI CIQOLP	1month after treatment
Valsara (2016) [33]	Individual CBT: 10 weekly sessions 1 hour each session (1) Behavioural activation (2) Cognitive restructuring (3) Didactic techniques Delivered by a doctoral-level nurse with CBT training	Non-directed counselling	depression	HADS	3months after treatment
Mehrotra (2019) [34]	Individual CBT: 10 weekly sessions 1 hour each session (1) psychoeducation (2) behavioural activation, (3) cognitive intervention (4) health behavioural modification Delivered by the therapists.	Sertraline	depression QoL	QIDS-C BDI-II Global quality of life scale	Not reported
Al saraireh (2018) [35]	Individual CBT: 7sessions 1 hour each session (1) Familiarization with CBT (sessions 1 and 2). (2) Active treatment (sessions 3 to 6), where we applied the specific CBT interventions. (3) Relapse prevention Delivered by nurses who had CBT expertise	Psychoeducation 7 sessions for one hour each time disease education, treatment education, stress management, relaxation techniques, positive thinking, optimism, deep breathing, problem-solving skills	Depression	HDRS	Not reported

*BDI* Beck depression inventory, *BDI-II* Beck depression inventory II. MINI: Mini International Neuropsychiatric interview, *HADS* Hospital anxiety and depression scale, *HDRS* Hamilton depression rating scale, *QIDS-C* Quick Inventory of Depressive Symptoms-Clinician-rated, *KDQOL-SF* Kidney disease and quality of life-short form, *QIDS-C* Quick inventory of depressive symptoms-clinician-rated, *CIQOLP* Chronic Ill Quality of Life Profile

### Results of study quality assessment

**Fig. 2:**
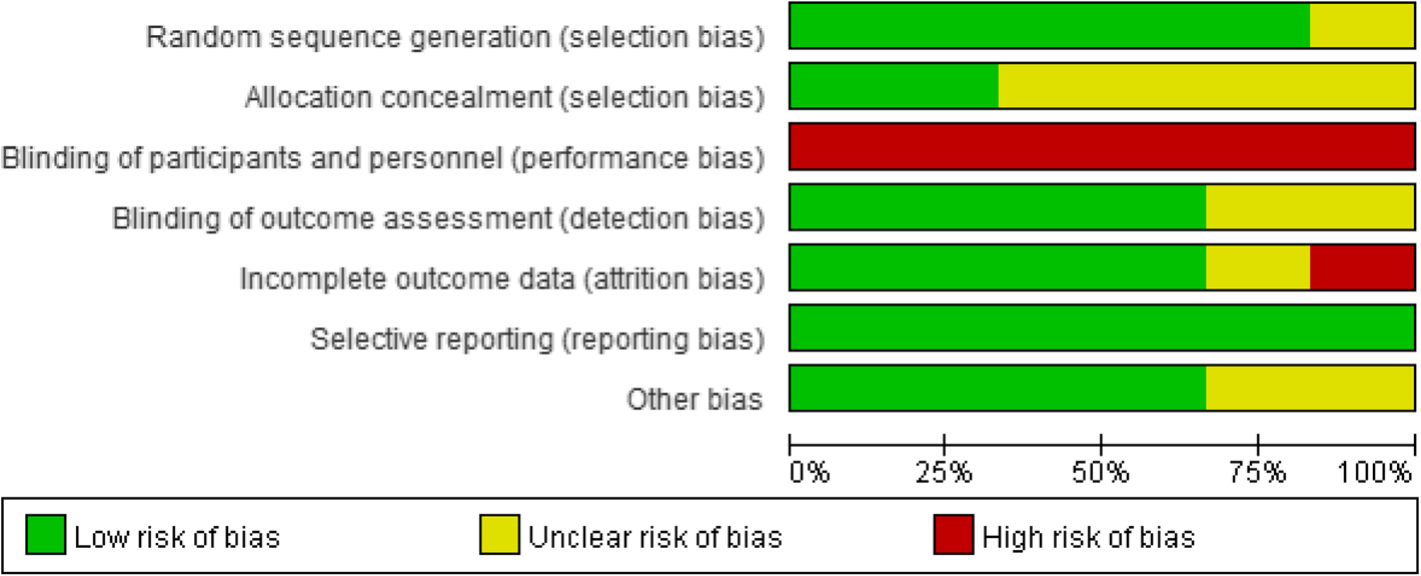
Risk of bias graph: review authors’ judgements about each risk of bias item presented as percentages across all included studies

**Fig. 3:**
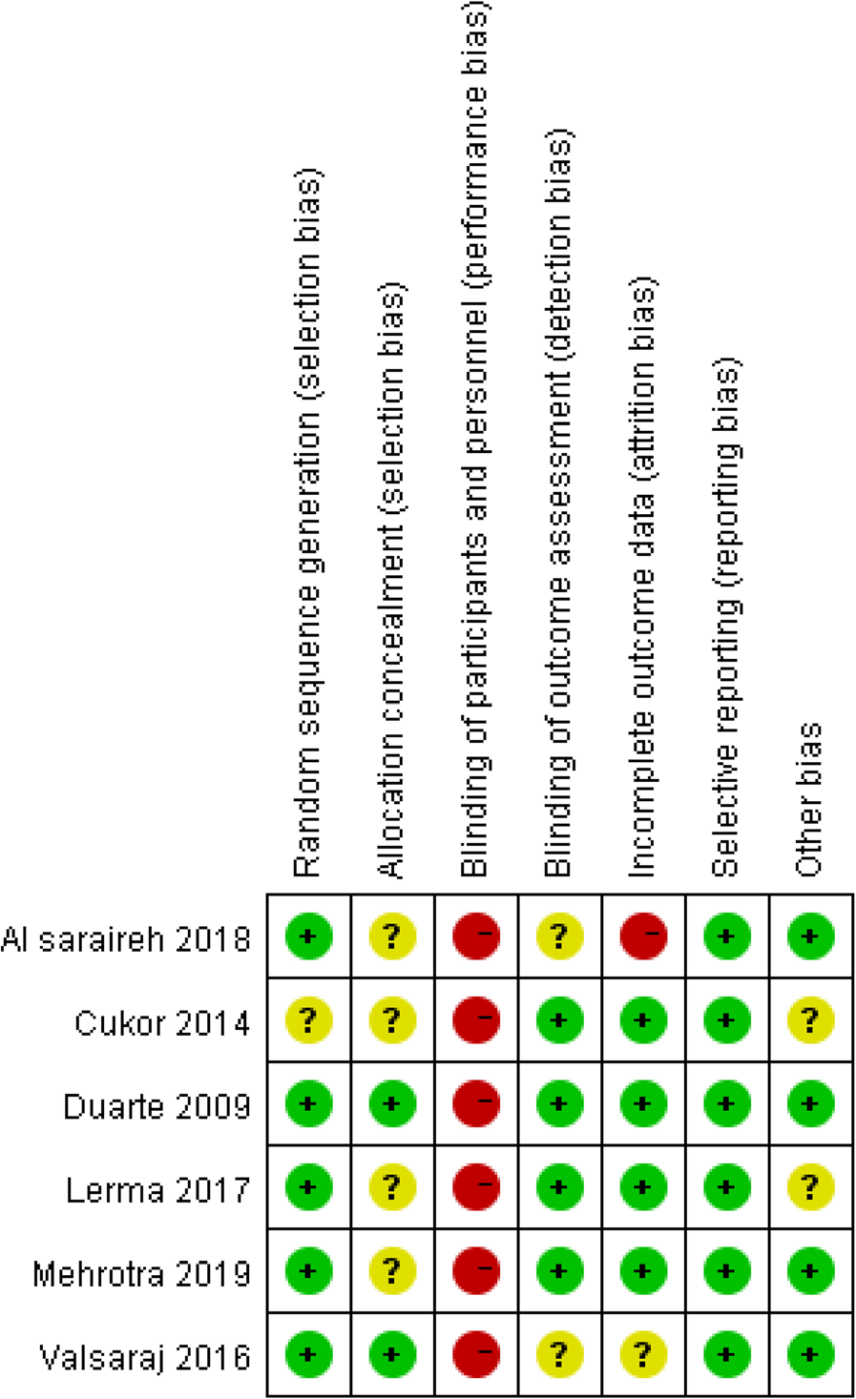
Risk of bias summary: review authors’ judgements about each risk of bias item for each included study. Detailed legend: Read the main text --Results of study quality assessment (Page 19–20)

Figure 2 and Fig. 3 below present a summary of the risk of bias across studies.

#### Random sequence generation

All studies were described as “randomised”, and five of the six studies reported adequate information about randomisation. However, one study [32] was rated as unclear because there were insufficient details about the methods of randomisation.

#### Allocation concealment

Four studies [30, 32, 34, 35] failed to state the detailed information of allocation concealment. Therefore, these four studies were rated unclear by default. Two studies [31, 33] used sealed envelopes to conceal the assignments, which in turn avoids selection bias. Hence, these two studies were rated as at low risks of allocation concealment.

#### Blinding of participants and personnel

Given the nature and method of implementation of CBT, it was impossible to keep the persons receiving or delivering the intervention or usual care blinded. Therefore, all studies were at high risk of performance bias.

#### Blinding of outcome assessment

In the six studies, four articles explicitly stated the blinding of outcome assessors [30–32, 34]. Hence, they were at low risk of detection bias. There was no description of the blinding of outcome assessment in the remaining two studies [33, 35]. Hence, the detection bias was rated as unclear in these two studies.

#### Incomplete outcome data

Four studies [30–32, 34] were rated as low risk of attrition bias due to the relatively low and balanced dropout rates, and clearly stated reasons. Valsara et al.’s study [33] failed to report the reasons for dropout. Therefore, the attrition bias of Valsara et al.’s study was considered as unclear. One study had higher attrition rates (CBT group was 21.6%, while 25.9% in the psychoeducation group) [35]. Therefore, Al saraireh et al.’s study [35] was rated as at high attrition risk.

#### Selective reporting

One trial protocol was published in Mehrotra et al.’s study [34]. All the outcomes were reported as planned. For the other five articles, selective reporting bias was not able to be assessed due to a lack of published protocols. Therefore, the methodologies and results sections of these five studies were carefully scanned to find incomplete data reports. All of the articles reported the pre-set outcomes. Hence, the rest of the five studies were rated as at low reporting bias.

### Effects of the intervention

The summary of the outcomes and effects of the interventions are elaborated in Table 3 below.

**Table 3:** Effect of intervention and control groups for HD on symptoms of depression and QoL at post-treatment and follow-up

Study ID	Time-point	Depression				QoL			
		Measure	Intervention	Control	MD/SMD (95% CI)	Measure	Intervention	Control	MD/SMD (95% CI)
Duarte (2009) [31]	post-treatment	BDI	14.1 (8.7)	21.2 (9.1)	MD: -7.1 (-10.88, -3.32)	KDQOL	Only sub-dimensions scores of the scale were reported		
	follow-up (6 mon)		10.8 (8.8)	17.6 (11.2)	MD: -6.8 (-11.07, -2.53)				
Cukor (2014) [32]	post-treatment	BDI-II	11.7 (9.8)	14.5 (8.5)	MD: -2.8 (-7.47,1.87)	KDQOL	115.3 (25.5)	110.6 (25.1)	SMD: 0.18(-0.33,0.70)
	follow-up (3 mon)		9.9 (8.5)	9.1 (6.5)	MD:0.8 (-3.03,4.63)		118.3 (27.7)	119.7 (24.7)	SMD: -0.05(-0.57,0.46)
	post-treatment	HAM-D	6.5 (6.8)	10.9 (5.4)	MD: -4.4 (-7.51, -1.29)	-	-	-	-
	follow-up (3 mon)		6.7 (5.8)	5.0 (4.3)	MD:1.7 (-0.87,4.27)	-	-	-	-
Lerma (2017) [30]	post-treatment	BDI	10.2 (8.2)	15.0 (10.9)	MD: -4.8 (-10.6,1.00)	CIQOLP	109.6 (21.1)	94.0 (21.0)	SMD: 0.73 (0.13,1.33)
	follow-up (1 mon)		7.1 (7.2)	14.7 (9.7)	MD: -7.6 (-12.7, -2.45)		112.5 (23.8)	91.3 (22.5)	SMD: 0.89 (0.28,1.50)
Valsara (2016) [33]	post-treatment	HADS	6.82 (1.86)	9.21 (2.69)	MD: -2.39 (-3.49, -1.29)	Not reported			
	follow-up (3 mon)		6.73 (1.53)	9.74 (2.71)	MD: -3.01 (-4.06, -1.96)				
Mehrotra (2019) [34]	post-treatment	QIDS-C	8.1 (5.1)	5.9 (4.5)	MD:2.2 (0.43,3.97)	GQOL	5.6 (5.0 to 6.2)	6.4 (5.8 to 7.0)	-
Al saraireh (2018) [35]	post-treatment	HADS	15.0 (5.5)	11.1 (2.3)	MD:3.9 (2.27,5.52)	Not reported			

#### CBT vs usual care

Three studies compared CBT versus usual care at post-treatment and follow-up.

##### Reduction in depressive symptoms

**Post-treatment**

**Fig. 4:**
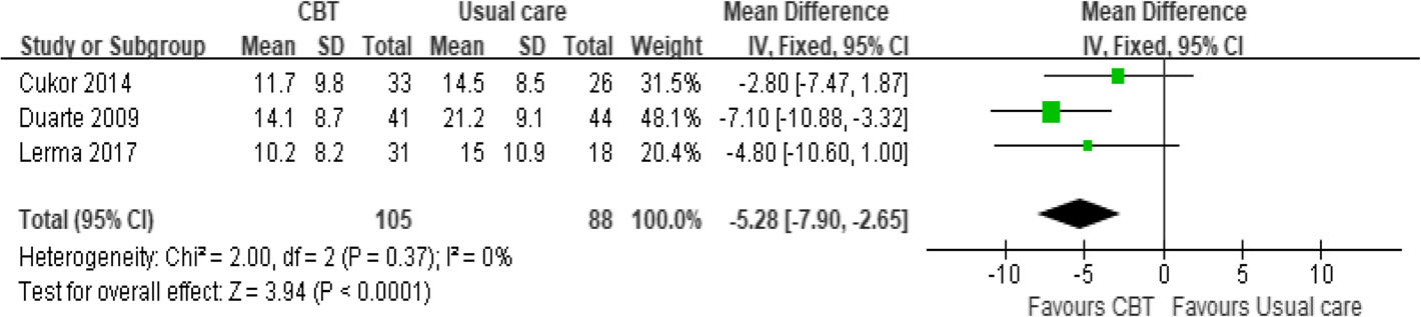
Forest plot of CBT vs usual care in the reduction of depressive symptoms after post-treatment. Detailed legend: Read the main text --Effects of the intervention (Page 22–23)

The meta-analyses of the three CBT versus usual care studies for depression are shown in Fig. 4. The CBT studies favoured the direction of the intervention, showing improvements in symptoms of depression (MD = − 5.28, 95% CI − 7.9 to − 2.65, *p* = 0.37).

**Fig. 5:**
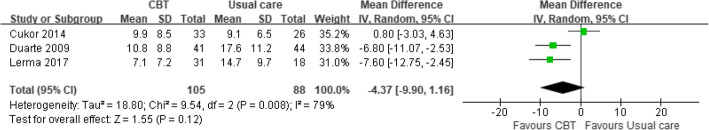
Forest plot of CBT vs usual care in the reduction of depressive symptoms after follow-up. Detailed legend: Lerma et al.’s [28] study reported the significant difference (MD = -7.6, 95%CI − 12.75 to − 2.45) between two groups during the 4 weeks follow-up after treatment. Similarly, in Duarte et al.’s [29] study, the difference between CBT compared with usual care was also be found during the 6 months follow-up after treatment (MD = -6.8, 95%CI − 11.07 to − 2.53). In contrast, in Cukor et al.’s [30] study, there was a non-significant effect in reducing the depression symptoms between the CBT and usual care during the 3 months follow-up

Lerma et al.’s study [30] conducted five weekly CBT sessions. The calculated MD was − 4.8 (95%CI − 10.6 to 1.00), meaning that the difference in depressive symptoms mean scores between the CBT and usual care was not statistically significant (Fig. 5). In Cukor et al.’s [32] and Duarte et al.’s [31] studies, they all conducted 12 weeks of CBT. Duarte et al.’s study demonstrated the significant differences in favour of CBT (MD = − 7.1, 95%CI − 10.88 to − 3.32). Upon a closer looking in Duarte et al.’s study and compared the data between baseline (Table 2 above) and post-treatment, the depression level gradually decreased from moderate depression to mild depression in CBT group (baseline:24.2 ± 9.7, post-treatment: 14.1 ± 8.7, *P*<0.001). Conversely, the patients in the usual care group stayed in moderate depression level after the treatment (baseline:27.3 ± 10.7, post-treatment: 21.2 ± 9.1, *P*<0.001).

However, Cukor et al. ‘s [32] study showed no difference between the CBT and usual care (MD = − 2.8, 95%CI − 7.47 to 1.87) (Fig. 4). A more in-depth look at the baseline and post-treatment depression scores, the depression level of both groups changed from moderate to mild depression (post-treatment in CBT group: 11.7 ± 9.8; post-treatment in usual care group: 14.5 ± 8.5). Additionally, Cukor et al.’s [32] study also used the HAM-D scale to test the effectiveness of CBT. Compared with the non-significant results measured by BDI, the results measured by HAM-D scales showed a significant difference in favour of CBT compared with usual care (MD = -4.4, 95%CI − 7.51 to − 1.29). Furthermore, the depression level reduced significantly from moderate depression to normal condition in the CBT group, while the participants in the control group stayed a mild degree of depression using the HAM-D tool.

**Follow-up**

The meta-analyses of the three CBT versus usual care studies for depression are shown in Fig. 5. The CBT studies favoured the direction of the intervention, showing improvements in symptoms of depression (MD = − 4.37, 95% CI − 9.90 to 1.16, *p* = 0.008). Statistically significant heterogeneity was found in this analyse (I^2^ = 79%).

Three studies reported the depressive scores at follow-up (Fig. 5). Lerma et al.’s [30] study reported the significant difference (MD = -7.6, 95%CI − 12.75 to − 2.45) between two groups during the 4 weeks follow-up after treatment. Similarly, in Duarte et al.’s [31] study, the difference between CBT compared with usual care was also be found during the 6 months follow-up after treatment (MD = -6.8, 95%CI − 11.07 to − 2.53). In contrast, in Cukor et al.’s [32] study, there was a non-significant effect in reducing the depression symptoms between the CBT and usual care during the 3 months follow-up. (Fig. 5).

**Improvement in QoL**

Three studies demonstrated QoL outcomes between CBT with usual care. Duarte et al.’s [31] study stated that CBT had a positive effect of improving the mental component summary in the KDQOL scale (*P*<0.001 in the CBT group, *P* = 0.451 in usual care group), whilst the difference in physical component summary in the KDQOL scale was not significant (*P* = 0.577 in the CBT group, *P* = 0.604 in the control group).

Lerma et al.’s [30] study showed a significant difference between the CBT and usual care on QoL at post-treatment and 5 weeks follow-up (SMD = 0.73, 95%CI 0.13 to1.33; SMD = 0.89, 95%CI 0.28 to1.50). In contrast, in Cukor et al.’ [32] study, no statistically significant differences were found at post-treatment and follow-up.

#### CBT vs non-directed counselling

One study (67 participants) contributed to this outcome [33]. Compared to baseline, the two groups all decreased depression level from moderate to mild. Nevertheless, the difference in depression scores between the CBT group and the non-directed counselling was significant, favouring CBT. (MD -2.39, 95%CI − 3.49 to − 1.29). Similarly, there was also a significant difference (MD -3.01, 95%CI − 4.06 to − 1.96) after 3 months of follow-up. This study did not investigate the QoL outcome at post-treatment or follow-up.

#### CBT vs antidepressant

Mehrotra et al.’s [34] study (114 participants) compared the effectiveness between CBT and sertraline, and the depression symptoms were measured by QIDS-C. The two groups all showed significant effects in reducing depressive symptoms from moderate to mild. However, the results demonstrated that sertraline groups were more effective than CBT in reducing depressive symptoms immediately post-treatment (MD 2.2, 95%CI 0.43 to 3.97). The follow-up data of depressive symptoms was not reported. Regarding the QoL, the difference in QoL improvement between the CBT group and sertraline group was non-significant (Effect estimate with 95% CI: − 0.6 (− 0.2 to 1.4)).

#### CBT vs psychoeducation

Only Al saraireh et al.’s [35] study (105 participants) reported that psychoeducation reduced the HAM-D score significantly compared to CBT (MD 3.9, 95%CI 2.27 to 5.52). Compared to baseline, the severity of depression in the psychoeducation group decreased from severe to moderate, while the severity of depression in CBT group did not change. The change of depression scores at follow-up and QoL were not reported in their study.

## Discussion

### Summary of the main findings

All studies showed that depressive symptoms improved with CBT. Upon a closer look, the results demonstrated a beneficial effect of CBT on depressive symptoms and QoL when compared to usual care and non-directive counselling. It also stated that CBT was less effective than sertraline and psychoeducation in improving depressive symptoms.

## Discussion of the main findings

### Depression

#### CBT vs usual care

CBT seems to be more effective than usual care in alleviating depression. As mentioned before, three studies compared CBT with usual care, and they were varied in the quality of the evidence and results. Duarte et al.’s [31] study had the least risk of bias among these three studies (only had performance bias, which was unavoidable in conducting CBT). Given the strong evidence from Duarte et al.’s study, CBT appears to more effective than usual care in improving depressive symptoms.

Due to the sparse experiments on this topic, globally, there is no specific guidance of depression in HD patients. However, the finding of the present review is relatively consistent with the NICE guideline [18] on chronic disease patients with depression. This guideline recommends CBT for mild to moderate depression patients with a chronic illness condition [18]. Similarly, this finding is in line with the systematic review [19] indicating that CBT was more effective than usual care in heart failure patients with depression.

However, in the current review, it seems that HD patients with depression did not benefit from short-term CBT. In Lerma et al.’s [30] study, after 5 weeks CBT, the depressive score between the two groups did not show statistically significant difference. The possible reason might be that depression is a chronic condition; patients could not recover with limited psychological treatments. Likewise, NICE guidelines [18] also suggest that nine to 12 weeks CBT were needed for chronic disease patients with depression. However, the result of Lerma et al.’s (2014) study needs to be interpreted with caution due to the small sample size and relatively low quality of the evidence.

Interestingly, in the present review it was also found that CBT has a long-term sustainable effect among HD patients with depression. In Duarte et al.’s [31] study, at 6 months follow-up after the treatment of CBT, the depression scores decreased in CBT group and showed a significant difference between the comparison and intervention groups. This point is also supported by Cuijpers, Hollon [36]. The possible reasons for this effect could be explained in that patients in CBT groups are taught the skills and knowledge to identify maladaptive thinking and deal with the depressive symptoms. Since the patients were equipped with the coping strategies, they could take preventative methods to alleviate depressive symptoms [37]. Indeed, one of the aims of CBT is to empower clients to become their own therapist [17]. In that way, CBT could help patients prevent depression recurrence [38].

#### CBT vs counselling

In the present review, one study showed that CBT was more effective than non-directive counselling at post-treatment and 3 months of follow-up in HD patients [33]. The possible reasons for this result might be the different strategies used between CBT and counselling. CBT is task-oriented, focusing on changing the clients’ thinking and behaviour patterns, and finding solutions to the practical issues. In contrast, counselling is less directive. Counsellors use active listening and empathetic attitude strategies to help the patients to understand themselves better [39]. Valsara et al.’s [33] result supports the statements of NICE guidelines for depression in adults [18]. In this guideline, CBT is recommended as a frontline treatment, while counselling is suggested as a second-line intervention.

However, in recent years, a growing number of studies suggest that CBT and counselling have comparable effects [40, 41]. Therefore, it is unknown whether the recommendations of NICE guidance would be revised based on these current studies. As the number of studies on this topic was sparse, and the quality of Valsaraj et al.’s [33] study was not high, there is no firm conclusion for these two therapies. Hence, better-designed RCTs which improve on the methodology used by Valsaraj et al.’s study are needed in the future. However, evidence-based medicine is not only about the effectiveness of the intervention but also the preferences of the patients where possible [42]. Therefore, further studies could conduct not only quantitative studies to investigate the effectiveness of these two therapies but also qualitative research to explore the preferences and experiences of HD patients in these two kinds of psychotherapies.

#### CBT vs sertraline

It is noteworthy that, in the present review, the newest study conducted by Mehrotra et al. [34] reported that sertraline was slightly more effective than CBT in HD patients with moderate depression. Mehrotra et al.’s [34] study had a relatively high methodological quality. The multicentre design could balance the confounding factors, promoting generalisation. Moreover, compared to other studies in this review, the depressive symptoms in their studies are measured by clinician-rated validated depression scale. This could increase the reliability of the outcome measurements.

This finding is consistent with an RCT, which compared the effectiveness of CBT with sertraline in diabetes patients with depression [43]. In comparison to CBT, the rapid therapeutic effect is the most advantageous to antidepressants. However, compared to diabetes patients, the safety of the antidepressants should be emphasised among HD patients due to their limited renal function and the possibility of drug-drug interactions. Indeed, in Mehrotra et al.’s [34] study, the rates of adverse events were higher in the sertraline group. Therefore, for moderate depressive HD patients, both treatments could be considered, while the pharmacological therapies need to be taken into account carefully.

In addition, for HD patients with severe depression, the combination of CBT with antidepressants is worthy to further investigation. According of NICE (2009) [18], the guideline suggests that CBT with antidepressants can be utilised among severe depression patients with a chronic illness. However, most of the participants in the present systematic review were diagnosed with moderate depression. Hence, further study could investigate the efficacy of the combined function of CBT with antidepressants.

### QoL

Regarding QoL, CBT might have a positive influence in improving QoL. In the present review, four studies all showed that the QoL scores increased after the CBT when comparing to baseline QoL scores. However, comparing CBT with usual care and sertraline, different results were reported. Owing to the varied number of risk of biases of these four studies, the present author could not reach a convincing conclusion. Nevertheless, considering the methodological quality of Duarte et al.’s [31] study is higher than the other three studies, CBT could be suggested as an effective treatment in improving QoL among HD patients with comorbid depression.

### The applicability of evidence

The scope of the current systematic review was limited to adult HD patients with depressive symptoms. The literature on therapy for depression in paediatric HD was not reviewed. Furthermore, the majority of the adult patients were middle-aged population, which was inappropriate to apply the conclusion to the geriatric HD patients with depression. Lastly, most of the participants included in the present systematic review were assessed as having moderate depression. Hence, the conclusions of the current review may not be applicable to HD patients with severe depression.

### The applicability of CBT

Given that CBT could be considered as an efficient, safe treatment option for HD patients, renal department healthcare providers should consider CBT as a treatment option. According to Goh et al. (2018) [7], the CBT might hard to embed in standard care in terms of insufficient access for participants to this therapy and limited CBT providers [44]. Hence, the present author discussed the solutions of this issue in two ways, which is elaborated as follows.

Internet-based CBT can be considered as an effective treatment for HD patients with depression. In the present review, all studies used traditional face-to-face CBT. Barriers of face-to-face CBT include geographic distance, limited professional therapists and high cost of therapy [45]. To bridge these treatment gaps, Internet-based CBT has been proved as one kind of methods to resolve the barriers mentioned above. Furthermore, according to an updated meta-analysis conducted by Carlbring et al. [46], internet-guided CBT and traditional face-to-face CBT have equivalent effects. However, for HD patients with comorbid depression, there was an absence of evidence which used internet-based CBT. Therefore, further study could investigate this type of CBT in HD patients.

Nurses can be considered as deliverers of CBT. Generally, CBT is conducted by professional therapists or psychologists. Interestingly, one study conducted in the US after hurricanes Katrina and Rita demonstrates that CBT may not need to be performed by psychologists. In their research, twenty-two social workers used CBT to care ESRD patients after the disaster. The depressive symptoms were significantly improved after the therapy [47]. Truly, in the present review, two of the included studies showed that the CBT which was conducted by nurses also had a promising effect on decreasing depression scores. Likewise, an RCT with 279 chronic obstructive pulmonary disease patients with diagnosed anxiety, a nurse-led CBT has been proved to be a clinically and cost-effective treatment to alleviate anxiety symptoms [48]. Therefore, further study could investigate the effectiveness of nurse-led CBT in HD patients.

### Strengths and limitation of this systematic review

Only HD patients diagnosed with depressive symptoms were included in the present review. This is inconsistent with the previous three relevant systematic reviews [22, 23, 49] which failed to include participants diagnosed with depressive symptoms at baseline. The number of included studies was decreased due to this rigorous criterion. Nevertheless, the conclusions of the present review serve the most relevant population.

Only six RCTs with 479 participants were included in the current systematic review; the handful quantity of studies and small sample size limited generalisation. Secondly, the diagnostic criteria of depression, the definition of CBT components, format, duration, as well as the outcome measurements were varied in included studies. Thirdly, the quality of the included studies was varied. Only one study was rated as low risk of bias in most of the domains. Therefore, firm conclusions could not be identified due to the reasons above.

Fourth, most of the outcome measurements (depression and QoL) were self-reported questionnaires, which involved patients’ subjective feeling; this may also produce biases. In addition, publication bias might be generated due to merely English articles were included in the present review. Lastly, there were insufficient studies that investigated the long-term maintained effects of CBT. Only one study assessed the depressive symptoms and QoL at 6 months follow-up. Therefore, the long-lasting effect of CBT was unknown.

### Implications for practice

Depression screening and early intervention of depression might be essential in routine HD nursing. In the current review, the present author found that most of the included patients had moderate depression at baseline, while the proportion of mild depression patients was small. This condition indicates that healthcare providers need to find approaches to prevent depressive symptoms from deteriorating in the early stage of depression. Hence, screening and integrating the knowledge and skills of CBT with patients’ education might be an effective way to improve HD patients’ well-being.

### Implications for future research

At present, the quality and number of studies investigated in this field were insufficient. Therefore, more rigorous studies comparing the CBT with usual care and other treatments (for example, antidepressant) in HD patients with depression are needed in the future. In terms of the methodological quality or the existing evidence, future studies can focus on recruiting larger sample size, utilising allocation concealment and recruiting blinded outcome assessors to improve the quality of the studies. In regard to the gaps of the present review, future research can work toward the different approaches in CBT among HD patients with depression, such as internet-based CBT, CBT combined with antidepressants or nurse-led CBT. Additionally, more studies should focus on the long-term effects of CBT on depressive symptoms and QoL.

HD patients diagnosed with depression could be investigated in the future. Generally, depression should be diagnosed by professionals according to the Diagnostic and Statistical Manual of Mental Disorders, Fifth Edition (DSM-5). In the present review, none of the participants was diagnosed with depression according to DSM; most of them are screened by different depression questionnaires. Duarte et al’ s research used the MINI International Neuropsychiatric Interview to screen out the participants instead of depression questionnaires. However, MINI is applied to meet the need for a short but accurate structured psychiatric interview for multicentre clinical trials and epidemiology studies and to be used as the first step in outcome tracking in non-research clinical settings [50, 51]. Thus, MINI should not be used to officially diagnose depression. Given this status, the present author suggests that researchers could pay attention to this type of person.

## Conclusions

In summary, CBT has shown an encouraging effect on depressive symptoms and mental summary of QoL among HD patients with depressive symptoms. Twelve weeks of intervention can be recommended in HD clinical practice. However, due to the mixed quality and small quantity of the existing studies, firm conclusions were prevented.

## Supplementary information

**Additional file 1.** PRISMA Checklist for the present systematic review.

**Additional file 2.** The electronic search strategy from.

**Additional file 3.** Search result from CINHAL.

**Additional file 4.** Characteristics of excluded studies.

## Data Availability

Not applicable since no new data involved.

## Abbreviations

ESRD: End Stage Renal Disease
HD: Haemodialysis
CBT: Cognitive Behavioural Therapy
QoL: Quality of life
BDI: Beck Depression Inventory
HDRS: Hamilton Depression Rating Scale
MINI: Mini International Neuropsychiatric Interview
HADS: Hospital Anxiety and Depression Scale
QIDS-C: Quick Inventory of Depressive Symptoms-Clinician-rated
MD: Mean Difference
CI: Confidence Intervals
