# Supplementary material for: The effects of cognitive behavioural therapy on depression and quality of life in patients with maintenance haemodialysis: a systematic review
Source: BMC Psychiatry. 2020 Jul 14;20:369. doi: 10.1186/s12888-020-02754-2 (PMC7362428; doi:10.1186/s12888-020-02754-2)
Supplement: Supplementary file 2 — Additional file 2. The electronic search strategy from. [file 12888_2020_2754_MOESM2_ESM.docx]

**Additional file 2. Search strategy**

|  | **Population** | **Intervention** | **Outcome** |
| --- | --- | --- | --- |
| OR | Haemodialysis | Cognitive behavio* therapy | Depression |
| OR | Hemodialysis | Cognitive behavio* intervention | Depressive |
| OR | Dialysis | CBT | Depressed |
| OR | Maintained hemodialysis | Cognitive therapy | Depressive disorder, major |
| OR | Renal dialysis | Behavioural therapy | Dysthymia |
| OR | HD | Psychosocial | Melancholia |
| OR | MHD | Psychological | Psychological disorder |
| OR | End-stage renal disease | Psychotherapy | quality of life |
| OR | Kidney failure, chronic |  |  |
|  | 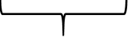 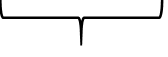  AND AND | | |
