# Supplementary figures and images for: The effects of cognitive behavioural therapy on depression and quality of life in patients with maintenance haemodialysis: a systematic review
Source: BMC Psychiatry. 2020 Jul 14;20:369. doi: 10.1186/s12888-020-02754-2 (PMC7362428; doi:10.1186/s12888-020-02754-2)

**Additional file 3. Search results from CINHAL.**


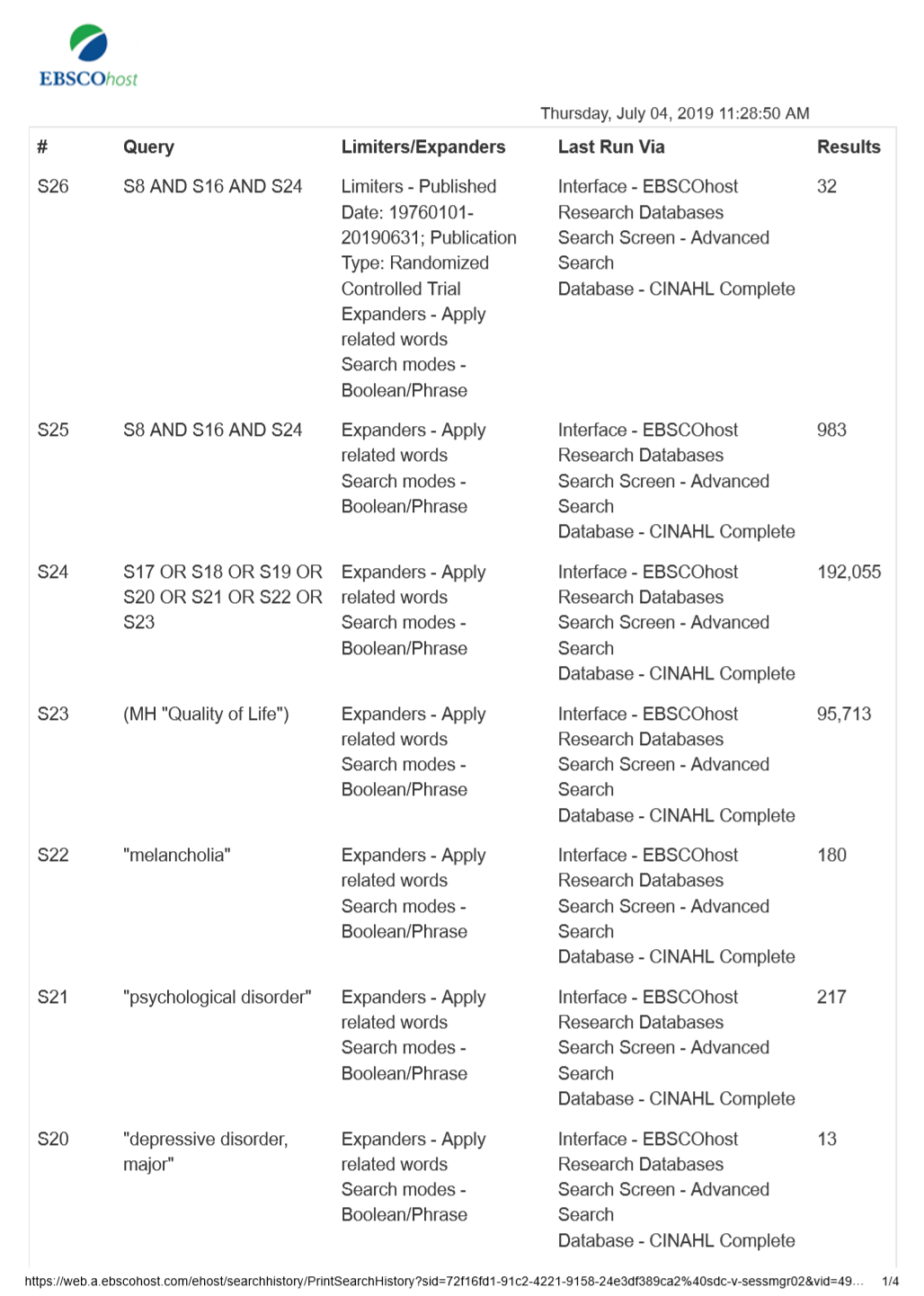


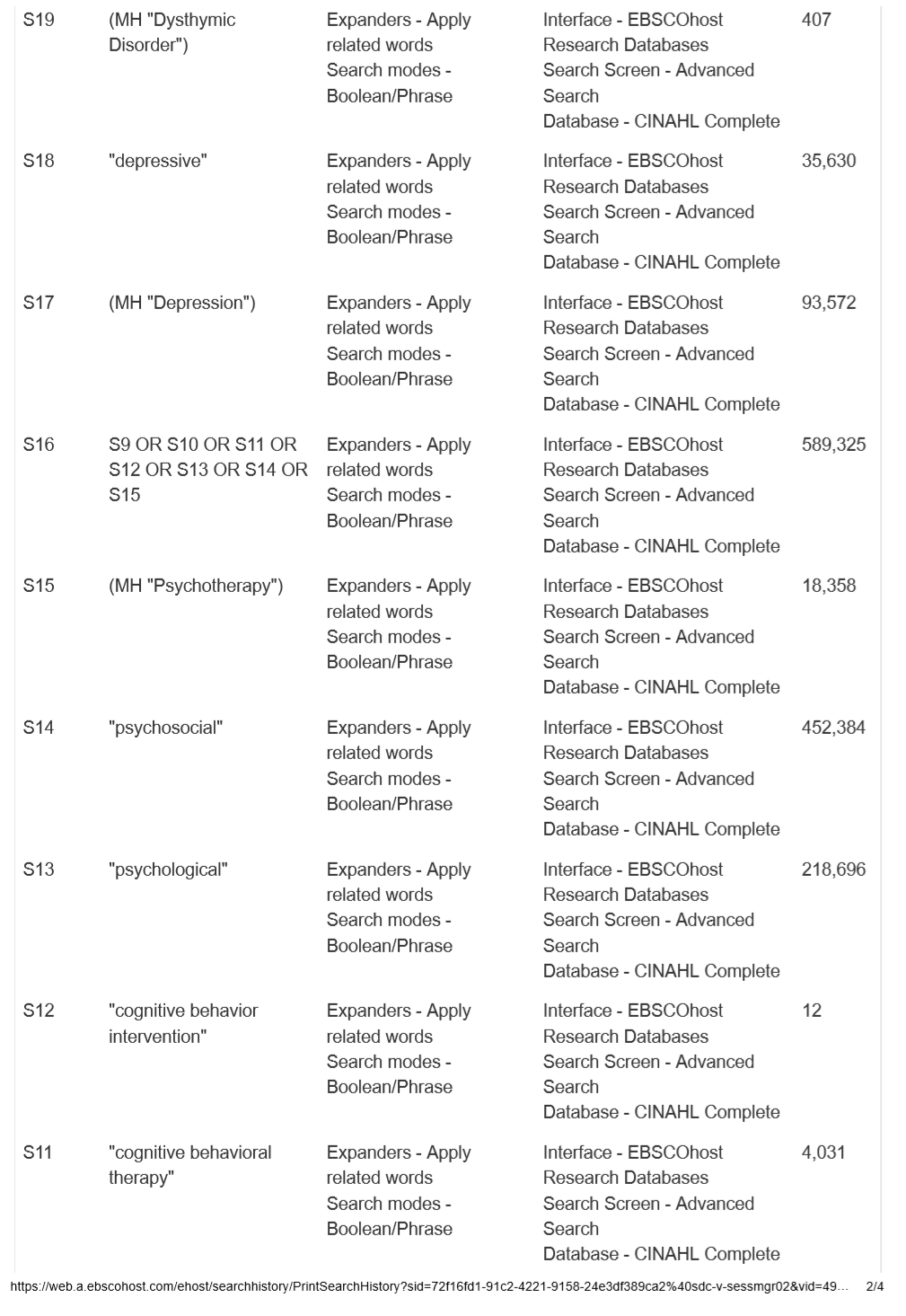


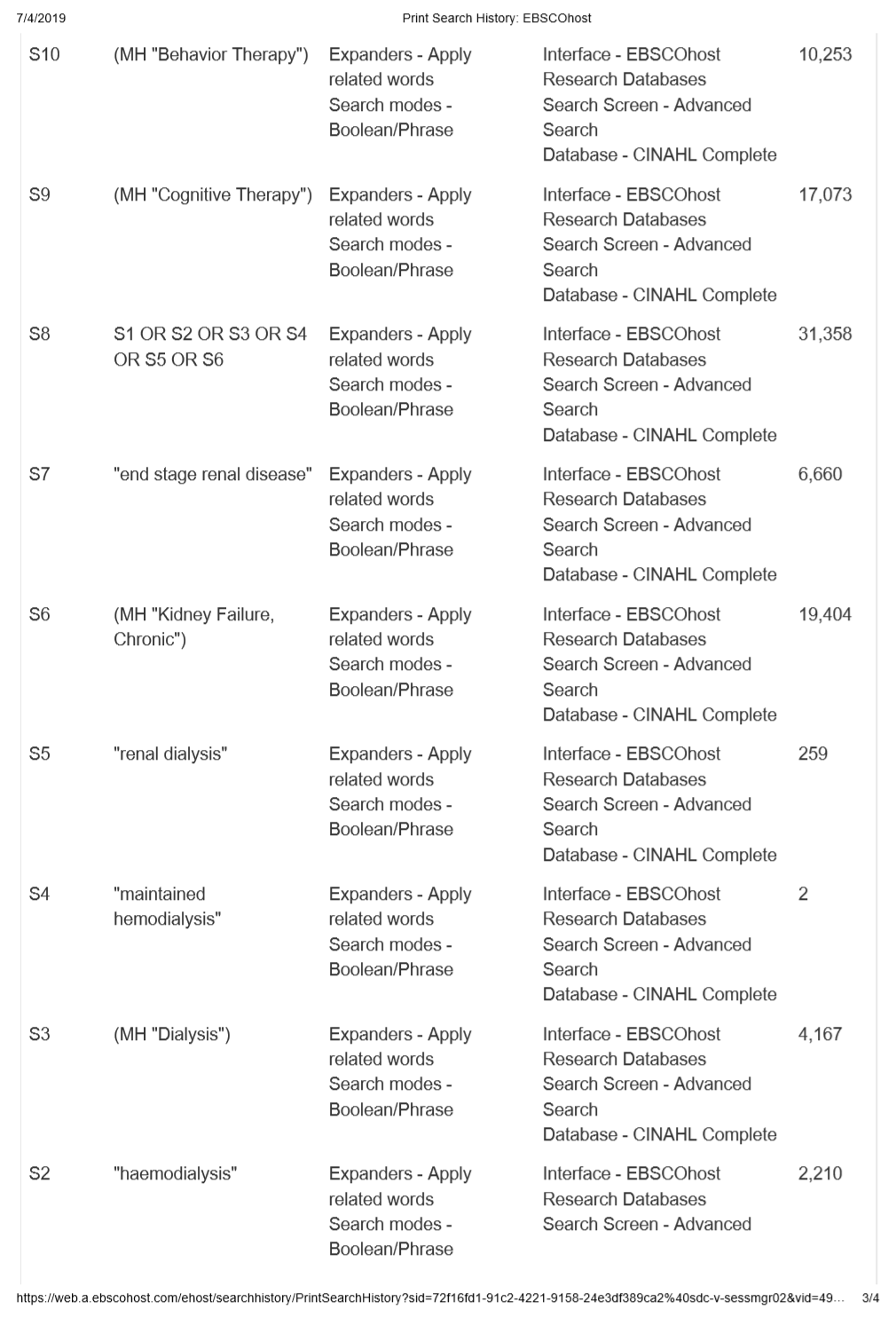


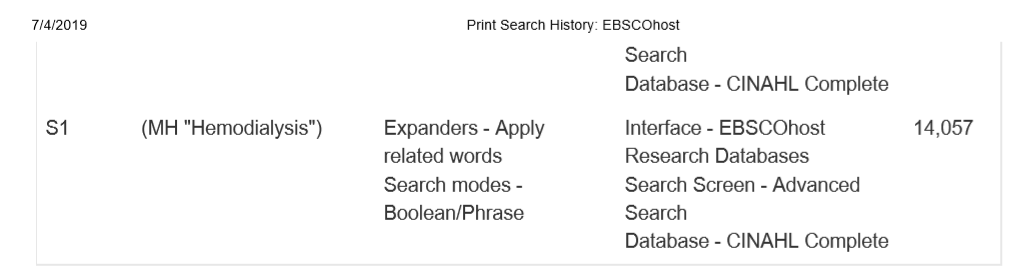

Supplement: Supplementary file 3 — Additional file 3. Search result from CINHAL. [file 12888_2020_2754_MOESM3_ESM.docx]
