# Supplementary material for: The effects of cognitive behavioural therapy on depression and quality of life in patients with maintenance haemodialysis: a systematic review
Source: BMC Psychiatry. 2020 Jul 14;20:369. doi: 10.1186/s12888-020-02754-2 (PMC7362428; doi:10.1186/s12888-020-02754-2)
Supplement: Supplementary file 4 — Additional file 4. Characteristics of excluded studies. [file 12888_2020_2754_MOESM4_ESM.docx]

**Additional file 4**. Characteristics of excluded studies (ordered by study ID)

| **Study ID** | **Reason for exclusion** |
| --- | --- |
| Chan et al. (2016) | Wrong design - not an RCT. |
| Chen et al. (2011) | Wrong population-HD patients without diagnosed depressive symptoms |
| Hedayati et al. (2016) | Wrong design –a primary study (RCT) protocol |
| Hou et al. (2014) | Wrong population-HD patients without diagnosed depressive symptoms |
| Hudson et al. (2016) | Wrong design - not an RCT, a self-management protocol |
| Kusztal et al. (2010) | Wrong design - not an RCT, a report only. |
| Lii et al. (2007) | Wrong population-HD patients without diagnosed depressive symptoms |
| Marvi et al. (2011) | Wrong population-HD patients without diagnosed depressive symptoms |
| Mehrtak et al. (2017) | Wrong design and population-not an RCT, HD patients without diagnosed depressive symptoms |
| Sharp et al. (2005) | Wrong population-HD patients without diagnosed depressive symptoms |
| Sohn et al. (2016) | Wrong design - not an RCT. |
| Solati et al. (2019) | Wrong population-HD patients without diagnosed depressive symptoms |
| Tsay et al. (2005) | Wrong population-HD patients without diagnosed depressive symptoms |
